# The Science and Practice of Medicine

**Published:** 1867-04

**Authors:** 


The Science and Practice of Medicine. By WILLIAM AITKEN, M.D., Edin., Prof. of Pathology in the Army Medical School; Corresponding Member of the Royal Imperial Society of Physicians of Vienna; of the Society of Medicine and Natural History of Dresden; and of the Imperial Society of Medicine of Constantinople; Late Pathologist attached to the Military Hospitals of the British Troops at Scutari; and Formerly Demonstrator of Anatomy in the University of Glasgow. In Two Volumes. From the Fourth London Edition, with Additions, by MEREDITH CLYMER, M.D., Late Professor of the Institutes and Practice of Medicine in the University of New York; Formerly Consulting Physician to the Philadelphia Hospital, etc. Philadelphia: LINDSAY & BLAKISTON. 1866. pp. 2069.

On the appearance of the first volume of the American edition of this work, we gave it a brief and commendatory notice. The second volume, comprising no less than 1114 pages, is now received from the American publishers, and completes the work. The two volumes embrace an aggregate of 2069 pages, and present the reader with a very full consideration of the science and practice of medicine as it exists at the present day. The author has not written it for the purpose of supplying a class of students with a text-book as cheaply as possible; nor as a labor-saving machine for indolent practitioners; nor yet because he had any special theories or favorite doctrines of his own to promulgate. The object of the author appears to have been, to present the profession with a full and well-digested summary of practical medicine as represented by the most eminent British writers and practitioners, not by any means, however, disregarding the medical literature of other countries. The notes and additions by the American editor, Dr. M. Clymer, are numerous and valuable. In the second volume, alone, they amount to over three hundred pages, embracing such topics as Gonorrhœal Rheumatism, Delirium of Inanition, Epileptiform Neuralgia, Auscultation, Sphygmograph, Chronic Pyæmia, Syphilitic Disease of the Liver, Addison's Disease, Cerebro-Spinal Meningitis, etc., etc. The American publishers have executed their task well, and the work is in all respects worthy of a place in the library of every medical practitioner. For sale by W. B. Keen & Co., 148 Lake St. Price $12.00.

